# The mediating role of metabolites between gut microbiome and Hirschsprung disease: a bidirectional two-step Mendelian randomization study

**DOI:** 10.3389/fped.2024.1371933

**Published:** 2024-08-27

**Authors:** Zhe Wang, Bingjun Gao, Xiao Liu, Aiwu Li

**Affiliations:** ^1^ Department of Pediatric Surgery, Qilu Hospital of Shandong University, Jinan, China; ^2^ Department of Neurosurgery, Qilu Hospital of Shandong University, Jinan, China

**Keywords:** Mendelian randomization, gut microbiome, Hirschsprung disease, metabolites, two-step, mediation proportion

## Abstract

**Background:**

Gut microbiome (GM) was observed to be associated with the incidence of Hirschsprung disease (HD). However, the effect and mechanism of GM in HD is still unclear. To investigate the relationship between GM and HD and the effect of metabolites as mediators, a bidirectional two-step Mendelian randomization (MR) study was conducted.

**Methods:**

The study selected instrument variables (IVs) from summary-level genome-wide association studies (GWAS). The MiBioGen consortium provided the GWAS data for GM, while the GWAS data for metabolites and HD were obtained from the GWAS Catalog consortium. Two-sample MR analyses were performed to estimate bidirectional correlations between IVs associated with GM and HD. Then, genetic variants related to 1,400 metabolite traits were selected for further mediation analyses using the Product method.

**Results:**

This study found that seven genus bacteria had a significant causal relationship with the incidence of HD but not vice versa. 27 metabolite traits were significantly correlated with HD. After combining the significant results, three significant GM-metabolites-HD lines have been identified. In the *Peptococcus*-Stearoyl sphingomyelin (d18:1/18:0)-HD line, the Stearoyl sphingomyelin (d18:1/18:0) levels showed a mediation proportion of 14.5%, while in the *Peptococcus*-lysine-HD line, the lysine levels had a mediation proportion of 12.9%. Additionally, in the *Roseburia*-X-21733-HD line, the X-21733 levels played a mediation proportion of 23.5%.

**Conclusion:**

Our MR study indicates a protective effect of *Peptococcus* on HD risk that is partially mediated through serum levels of stearoyl sphingomyelin (d18:1/18:0) and lysine, and a risk effect of *Roseburia* on HD that is partially mediated by X-21733 levels. These findings could serve as novel biomarkers and therapeutic targets for HD.

## 1 Introduction

Hirschsprung disease (HD) is a congenital disorder of enteric nervous system characterized by the absence of enteric ganglia in the distal part of the colon, leading to abnormal contractions (1). Until now, the only efficient treatment for HD is surgery to remove the aganglionic segment of the colon and reconstruct the healthy, innervated intestine to the anus (2). However, approximately one-third of HD patients still experience complications after surgery, including fecal soiling, obstructive symptoms, and Hirschsprung-associated enterocolitis (HAEC) (3). The cause of these complications is not well understood. Various studies have suggested possible mechanisms such as genetic mutation (4), dysfunction of the immune system in the gut (5), and an imbalance of the gut microbiome (6).

Gut microbiome (GM) is a distinct microbial ecosystem within the digestive system that plays a critical role in regulating human health and disease (7). The imbalance and dysfunction of microbial composition in GM, resulting from a metabolic disorder, can lead to several diseases (8). GM is deemed an extra “organ” with more genes than the host and is influenced by genetics and the environment (9). Several studies have shown that GM is associated with a range of illnesses, such as diabetes (10), cardiovascular disorders (11), neurological diseases (12), and immune diseases (13). Genome-wide association studies (GWAS) have challenged the view that the gut microbiota is solely an environmental factor (14). However, the extent of its genetic influence remains a topic of debate.

In order to investigate the potential mediation effect that could bridge the GM to the incidence of HD, metabolites are considered. Metabolites refer to tiny molecules produced either as a result or at the end of various metabolic reactions (15). Multiple factors, such as genetics, lifestyle, disease, and GM, can impact metabolite levels in the body (16, 17). The changes in metabolites can impact the incidence of disease. The high heritability of metabolites makes them convenient to study and potential therapeutic targets for disease (18). Based on these findings, we hypothesize that metabolites mediate the effect of GM on HD. However, it is difficult to establish the causal relationship and avoid confounding bias in an observational study. Therefore, an innovative methodology should be adopted to investigate the causal relationship.

Mendelian randomization (MR) is an effective method that can avoid the confounding bias and evaluate the effect of exposures on outcomes (19). MR analysis assumes that during conception, random assignment of alleles to offspring follows Mendel's law of inheritance, mirroring the concept of a randomized controlled trial (20). Using single nucleotide polymorphisms (SNPs) associated with specific IVs as proxies for exposures, MR analysis can assess the causal effects between exposures and outcomes (21). When the effect between exposures and outcomes is confusing, a mediation MR analysis can help to understand the cause better and identify intermediate variables that could be potential targets for intervention (22).

Our study aimed to investigate the relationship between GM and HD and explore the potential role of metabolites as mediators by conducting a bidirectional two-step MR analysis.

## 2 Materials and methods

### 2.1 Study design

**Figure 1:**
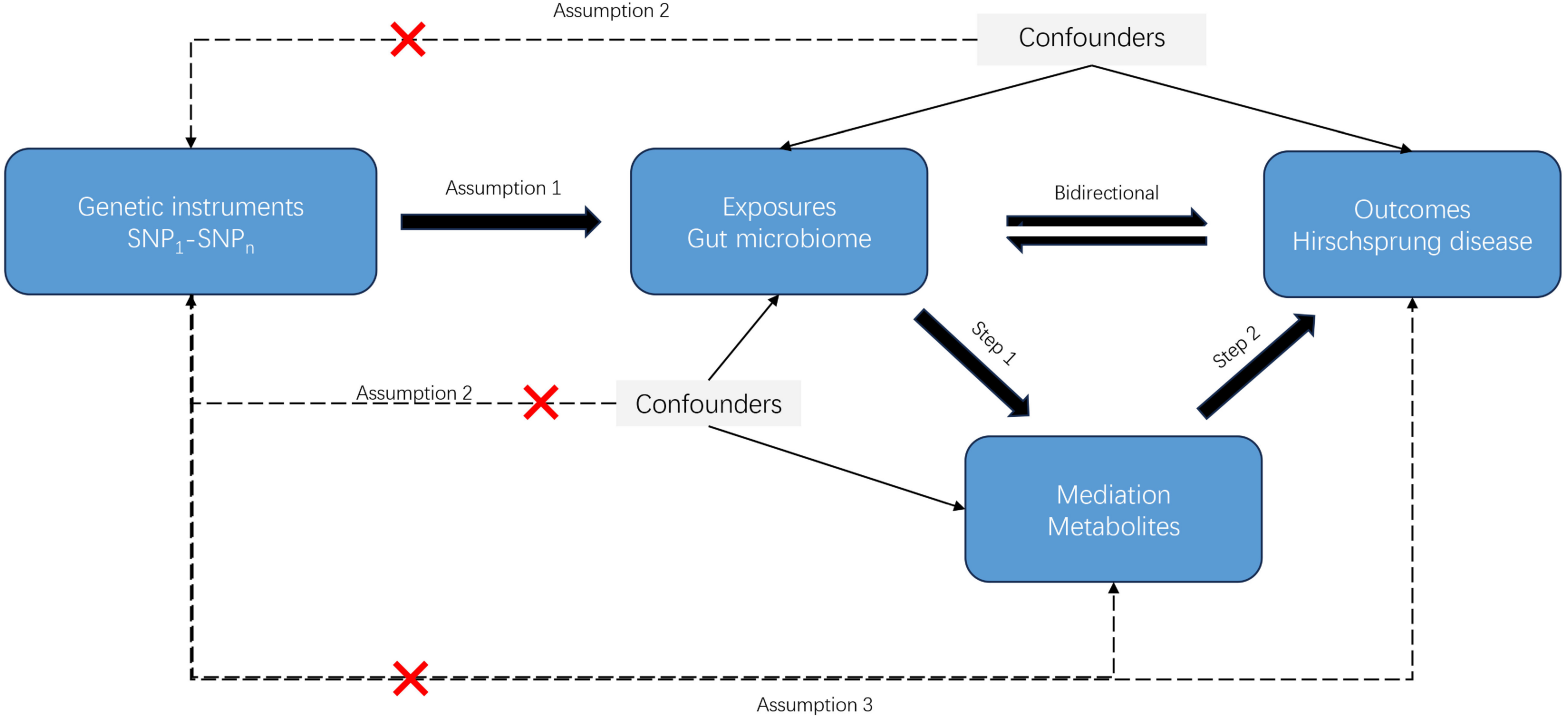
Study design of the MR analysis.

We used summary-level GWAS data to explore the genetic relationship between GM and HD in an MR framework. The effect of an exposure on an outcome consists of both direct and indirect effect (22). Our research involved a two-step MR analysis to investigate the causal relationship between GM and metabolites, followed by an assessment of the causal impact of metabolites on HD. To calculate the mediation effects of metabolites, we used the Product method, as previously described in other literature (23). Three fundamental assumptions must be met when selecting IVs, similar to a conventional MR study (24). Firstly, SNPs should show a strong correlation with exposures and meet the criterion for genome-wide significance threshold. Secondly, the selected IVs should be independent of any confounding factors affecting the study results. Lastly, the IVs should only affect the outcome through exposures and ensure that other lines or routes are not responsible for the effect on the outcome. The design of our bidirectional two-step MR study was illustrated in Figure 1.

### 2.2 Data sources

From the largest GWAS database on GM, the MiBioGen consortium (https://mibiogen.gcc.rug.nl), we obtained the summary-level data for GM, including 16S rRNA fecal microbiome data from 18,340 individuals (25). In this cohort, genetic loci impacting the covariate-adjusted abundance of bacterial taxa were adjusted for age, sex, technical covariates, and genetic principal components (25). Using these genetic markers, we can estimate the causal relationship between GM and complex traits through MR approaches (26).

Summary-level GWAS data for 1,400 metabolite traits (1,091 metabolites and 309 metabolite ratios) were extracted from the Canadian Longitudinal Study on Aging (CLSA) cohort of 8,299 individuals (15). The study found associations between 690 metabolites at 248 sites and 143 metabolite ratios at 69 sites. By integrating metabolite-gene and gene expression data, the study identified 94 effector genes for 109 metabolites and 48 metabolite ratios (15).

The GWAS Catalog consortium (https://www.ebi.ac.uk/gwas) provided GWAS data for HD, including 170 HD patients and 4,717 controls of European ancestry as the exposures (27). The diagnosis of HD was made using the Tenth Revision of the International Classification of Diseases (ICD-10) codes (28). In this cohort, the SNP heritability of HD has been estimated to be approximately 88% based on GWAS studies (27). Moreover, they have used Lasso regression to develop a potential genetic predictor for HD (27).

We conducted our study using publicly available GWAS summary data that has been approved by relevant ethics and institutional review boards. Therefore, we did not require ethical approval for our study. Details of the GWASs data are shown in Table 1.

**Table 1:** Details of GWAS data in this MR study.

Trait	Consortium	Ethnicity	Sample size
GM	MiBioGen	European	18,340
Metabolites	CLSA	European	8,299
HD	GWAS Catalog	European	4,887

GWAS, genome-wide association study; GM, gut microbiome; HD, Hirschsprung disease; CLSA, Canadian longitudinal study on aging cohort.

### 2.3 Selection of instrument variables

Suitable SNPs for IVs are genetic variants that serve as proxies for exposures to investigate causal relationships at the genetic level in MR studies (29). The selection of IVs was based on several principles: First, since only a small number of SNPs met the statistical significance threshold of *p* < 5e-08 for GM, metabolites, and HD, different threshold values were set for each measure (30). For GM and metabolites, the genome-wide significance threshold was set at *p* < 1e-05, while for HD, it was set at *p* < 5e-06. Second, SNPs with an *r*^2^ greater than 0.001 within a 10,000 kb distance were excluded due to linkage disequilibrium (LD) (31). Third, through the PhenoScanner website (http://www.phenoscanner.medschl.cam.ac.uk/) and as well as the IEU database (https://gwas.mrcieu.ac.uk/), we removed SNPs associated with inflammatory bowel disease, malignant neoplasm of colon, and irritable bowel syndrome in order to minimize the impact of confounding factors. Fourth, any SNPs that had a palindromic structure were excluded automatically from the analysis. Lastly, we calculated the *F*-statistics to assess the genetic liability of their genetic instruments using the following formula (32):

(1) $$F=(\mathrm{beta}/\mathrm{se}{)}^{2}$$

(se: standard error). From traditional experience, when the F statistic is less than 10, we usually consider the instrumental variable used as a weak instrumental variable, which may produce a certain bias to the result (33). To avoid weak instrument bias, only SNPs with *F* statistics greater than ten were chosen for the study. SNP characteristics are listed in Supplementary Data Sheet 2.

### 2.4 Statistical analysis

To explore the causal relationship between GM and HD, a bidirectional two-sample MR analysis was conducted. Then, a two-step MR analysis was conducted to evaluate the potential mediating effect via metabolites in this causal relationship. The inverse-variance weighted (IVW) method was utilized as the primary analysis, considering it the most reliable when there were no signs of directional pleiotropy (34). In the absence of horizontal pleiotropy, the IVW test was used as the primary method for calculating the causal effect values to obtain unbiased estimates. Additionally, weighted mode (34), MR-Egger (35), weighted median (36), and simple mode (37) were used as complementary approaches to assess the consistency of the results.

The Cochran's Q method was utilized with a *p*-value lower than 0.05 indicating heterogeneity (38). Cochran's Q is a kind of heterogeneity statistic for the IVW model. If the Q statistic much larger than its degrees of freedom, this provides evidence for heterogeneity and invalid IVs. The MR-Egger regression analysis was used for identification of potential horizontal pleiotropy (39), while the MR pleiotropy residual sum and outlier (MR-PRESSO) analysis (40) was conducted to minimize possible confounding factors. To investigate the effect of a single SNP on causal associations, we conducted leave-one-out sensitivity tests by removing each SNP one at a time (41). Additionally, scatter plots and funnel plots were generated to assess the robustness of the MR results.

The mediation proportion of potential mediators in the total effect of genetically determined GM on HD risk was calculated using the Product method (22). The mediation proportions were determined using the following formula (42):

(2) $$P(\text{\%})\;=\;\left(\beta_{1}\times \beta_{2}\right)/\beta_{0}$$

P(%): the proportions of mediation; β_0_ stands for the total effect obtained from the primary analysis, β_1_ stands for the effect of GM on mediators, and β_2_ stands for the effect of mediators on HD.

The statistical analysis was conducted using R packages “TwoSampleMR” and “MRPRESSO” in R software (version 3.4.2, the R Foundation for Statistical Computing, Vienna, Austria). The findings were presented in a combined format of odds ratio (OR) and a 95% confidence interval (CI).

## 3 Results

### 3.1 Selection of instrument variables

After performing the clumping process for LD, 1,425 SNPs were identified as IVs associated with GM traits (*p* < 1e-05). We only considered the genus level data, which contains the most relevant information about the GM and overlaps with higher classification levels (43). For metabolites, we analyzed 33,571 SNPs, with each metabolite corresponding to an average of 24 SNPs. We selected 18 SNPs as IVs for HD (*p* < 5e-06). After harmonizing with the metabolites data, only nine SNPs were left. The average *F*-statistics for GM, metabolites, and HD were 21.73, 29.88, and 38.34, respectively. All *F*-statistics exceeded 10, indicating avoidance of potential instrumental bias. Finally, we removed the palindromic SNPs simultaneously. The details of selected SNPs are shown in Supplementary Data Sheet 2.

### 3.2 Bidirectional two-sample MR analyses of the total effect between GM and HD

**Figure 2:**
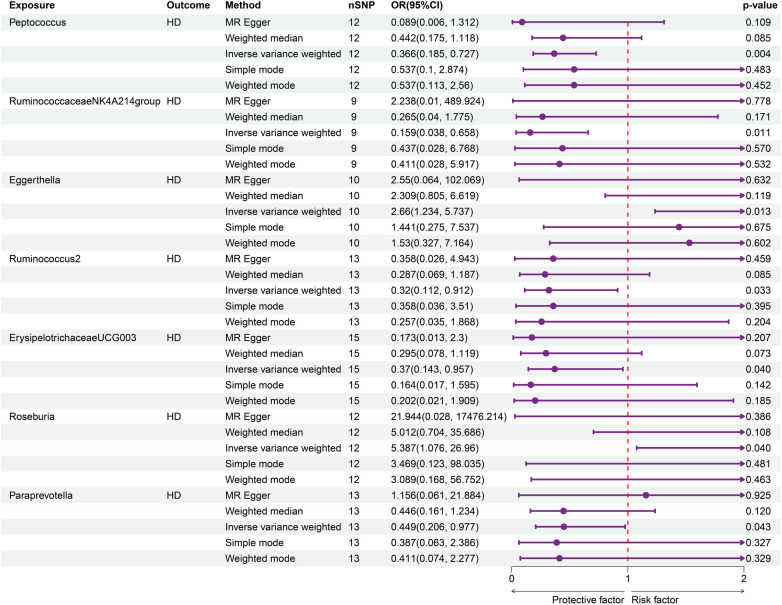
Forward MR analysis of GM on HD. Elevated Peptococcus (OR = 0.366, 95% CI = 0.185–0.727, *p* = 0.002), RuminococcaceaeNK4A214group (OR = 0.159, 95% CI = 0.038–0.658, *p* = 0.011), Ruminococcus2 (OR = 0.32, 95% CI = 0.112–0.912, *p* = 0.033), ErysipelotrichaceaeUCG003 (OR = 0.37, 95% CI = 0.143–0.957, *p* = 0.039), and Paraprevotella (OR = 0.449, 95% CI = 0.206–0.977, *p* = 0.043) were linked with decreased susceptibility to HD.

Seven genus bacteria were found to have a causal effect on an increased incidence of HD. Our forward MR analyses revealed that IVs associated with an elevated *Peptococcus* (OR = 0.366, 95% CI = 0.185–0.727, *p* = 0.002), *RuminococcaceaeNK4A214group* (OR = 0.159, 95% CI = 0.038–0.658, *p* = 0.011), *Ruminococcus2* (OR = 0.32, 95% CI = 0.112–0.912, *p* = 0.033), *ErysipelotrichaceaeUCG003* (OR = 0.37, 95% CI = 0.143–0.957, *p* = 0.039), and *Paraprevotella* (OR = 0.449, 95% CI = 0.206–0.977, *p* = 0.043) were responsible for decreased susceptibility to HD. The participants of *Eggerthella* (OR = 2.66, 95% CI = 1.234–5.737, *p* = 0.013) and *Roseburia* (OR = 5.387, 95% CI = 1.076–26.96, *p* = 0.04) brought a higher incidence of HD (Figure 2). Supplementary Table S1 provides a clear illustration of the causal effects of GM on HD.

**Figure 3:**
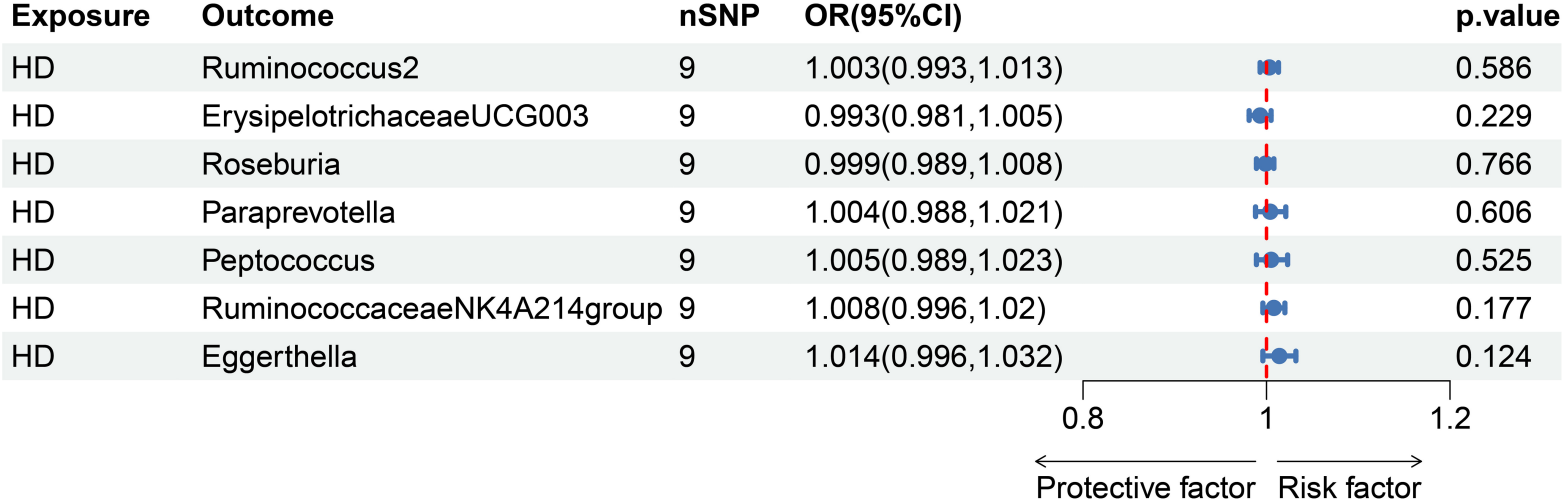
Reverse MR analysis of HD on GM.

On the other hand, the reverse MR analysis showed that there was no impact of genetic predisposition to HD on pre-identified GM traits. The results of IVW estimates are illustrated in Figure 3 and Supplementary Table S2.

### 3.3 Two-step MR analyses

**Figure 4:**
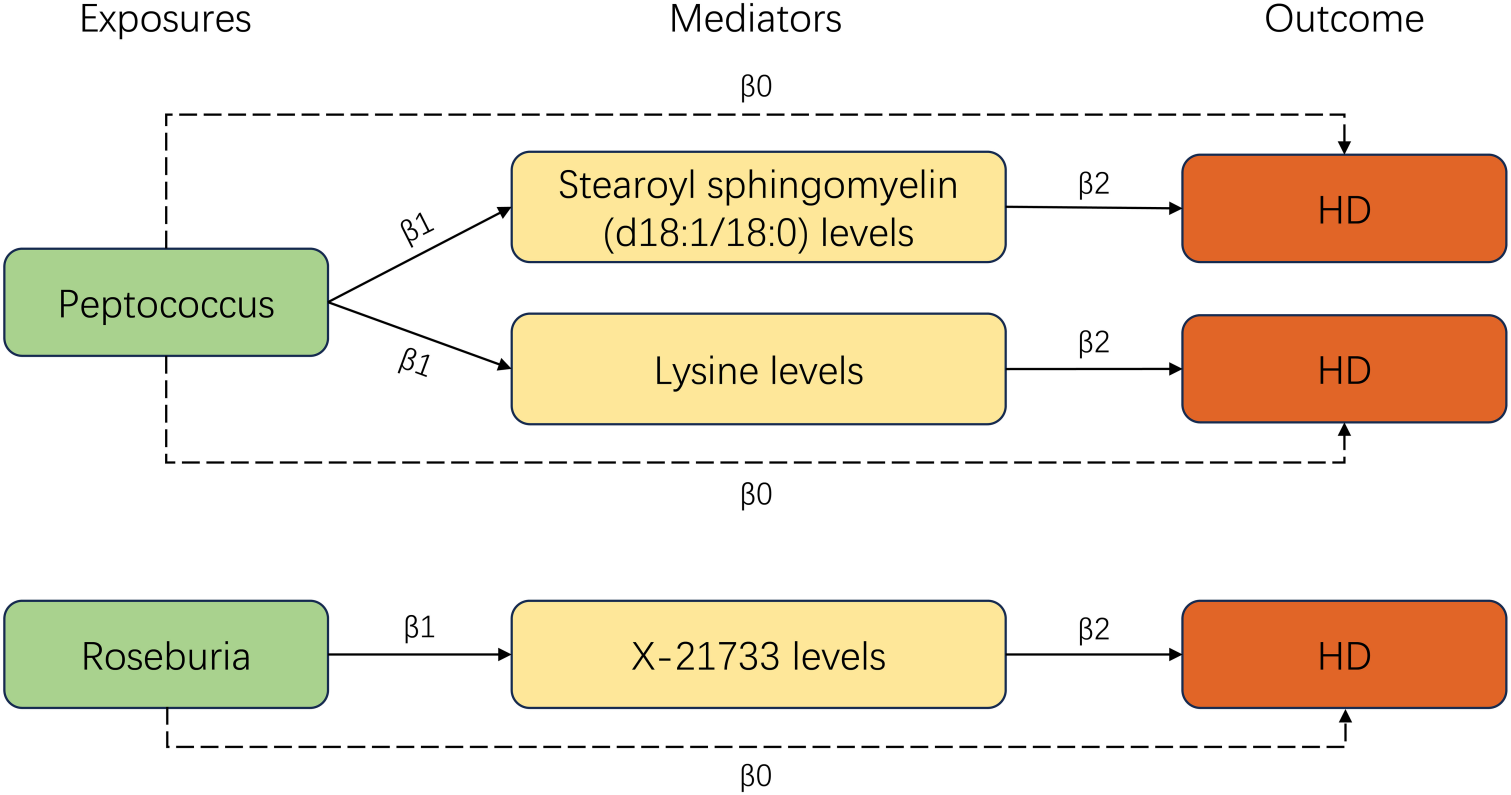
Three significant lines of the two-step MR analysis.

Our study utilized a two-step MR analysis approach. In the first step, we conducted a two-sample MR analysis to investigate the causal relationships between metabolites and the incidence of HD. This analysis revealed a significant link between 27 metabolite traits and HD (Supplementary Table S3). In the second step, we used the univariable MR method to investigate the relationship between the seven types of GM and 27 metabolite traits. Our findings identified three significant lines of evidence, including two types of bacteria and three metabolites. After combining these significant results, we obtained three significant GM-metabolites-HD lines (Figure 4).

#### 3.3.1 Causal effects of GM on metabolites

**Figure 5:**
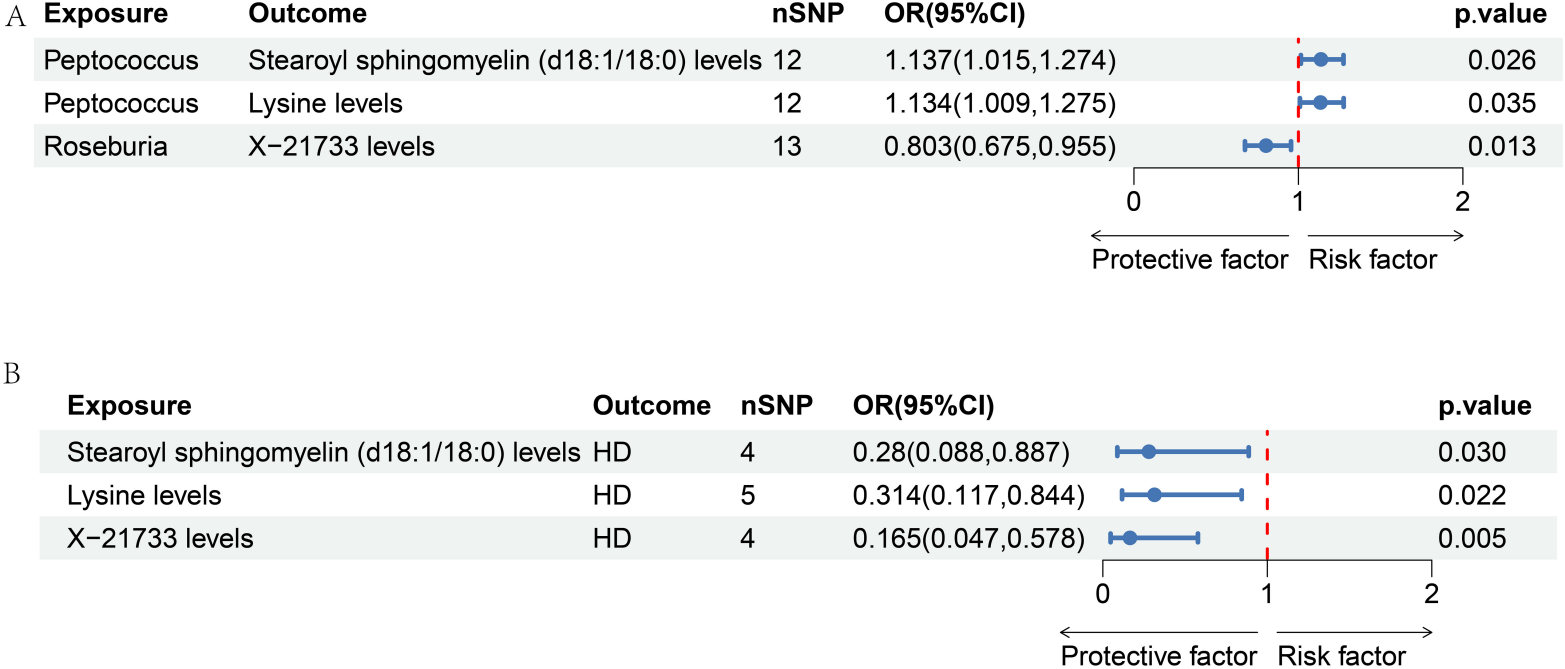
Forest map of two-step MR analysis of GM-metabolite-HD. **(A)** The causal effects of GM traits on metabolites using the IVW method.; **(B)** the causal effects of metabolites on HD, including stearoyl sphingomyelin (d18:1/18:0) levels (OR = 0.28, 95% CI = 0.088–0.887, *p* = 0.03), lysine levels (OR = 0.314, 95% CI = 0.117–0.844, *p* = 0.022), and X-21733 levels (OR = 0.165, 95% CI = 0.047–0.578, *p* = 0.005).

According to our MR analysis, *Peptococcus* had a positive correlation with stearoyl sphingomyelin (d18:1/18:0) levels (OR = 1.137, 95% CI = 1.051–1.274, *p* = 0.026) as well as lysine levels (OR = 1.134, 95% CI = 1.009–1.275, *p* = 0.035). We found a negative correlation between *Roseburia* and X-21733 levels (OR = 0.803, 95% CI = 0.675–0.955, *p* = 0.013). Figure 5A displays the causal effects of GM traits on metabolites using the IVW method. Details are shown in Supplementary Table S3.

#### 3.3.2 Causal effects of metabolites on HD

We observed that 27 metabolite traits were significantly correlated with incidence of HD (Supplementary Table S4). Three metabolite traits were identified to be the significant mediators, including stearoyl sphingomyelin (d18:1/18:0) levels (OR = 0.28, 95% CI = 0.088–0.887, *p* = 0.03), lysine levels (OR = 0.314, 95% CI = 0.117–0.844, *p* = 0.022), and X-21733 levels (OR = 0.165, 95% CI = 0.047–0.578, *p* = 0.005). The significant effect of metabolites on HD is illustrated in Figure 5B.

#### 3.3.3 Mediating effects of metabolites on GM-HD effect

In this two-step MR analysis, we identified the metabolites that could potentially mediate the causal effect of GM on HD. In the first step, we found that stearoyl sphingomyelin (d18:1/18:0) levels, lysine levels, and X-21733 levels could be affected by GM. In the second step, we observed that these same metabolites had a significant role in mediating the effect of GM on HD. We used the Product method to calculate the mediation proportion. In the *Peptococcus*-Stearoyl sphingomyelin (d18:1/18:0)-HD line, the Stearoyl sphingomyelin (d18:1/18:0) levels showed a mediation proportion of 14.5%, while in the *Peptococcus*-lysine-HD line, the lysine levels had a mediation proportion of 12.9%. In the *Roseburia*-X-21733-HD line, the X-21733 levels played a mediation proportion of 23.5%.

### 3.4 Sensitivity analyses

We conducted Cochran's Q analysis and found no significant heterogeneity in the estimates. We also detected the *p*-value associated with the MR-Egger intercept, and found no potential pleiotropic effects. Moreover, we utilized multiple estimating methods to evaluate the causal effects, and the scatter plots involving these estimating methods were shown in Supplementary Figure S1. The consistency results of the sensitivity analyses reinforced the causal reasoning of the primary analyses. Additionally, forest plots, funnel plots, and leave-one-out plots of each IV showed that the results of MR analysis were consistent and robust (Supplementary Figures S2–4).

## 4 Discussion

This study explored the causal relationship between GM and HD, and assessed the mediating effect of serum metabolite traits between GM and HD in a comprehensive bidirectional two-step MR analysis. Our study revealed that genetically variants in *Ruminococcus2*, *ErysipelotrichaceaeUCG003*, *Roseburia*, *Paraprevotella*, *Peptococcus*, *RuminococcaceaeNK4A214group*, and *Eggerthella* were significantly associated with the incidence of HD. The results did not indicate bidirectional causal relationship. Our two-step MR analyses revealed that *Roseburia* contributed to an increased risk of HD, which was partially mediated by lower levels of X-21733. As the original paper stated that X-21733 is an unknown molecule in serum (15), we have excluded it from further discussion. On the other hand, *Peptococcus* was found to be associated with a reduced risk of developing HD, partially mediated by levels of stearoyl sphingomyelin (d18:1/18:0) and lysine.

Studies conducted on animals and humans have confirmed a connection between the diversity of GM and HD. The human gastrointestinal tract commonly acts as a natural ecosystem and supplier of nutrients for microbiota. In return, microbiota aids in gut development, strengthen the immunity, and improve the defense mechanisms by generating different metabolites (44). Cheng et al. found decreased fecal microbiota alpha diversity in HD mice after microsurgical pull-through surgery on Ednrb knock-out mice (45). A study on children with HD showed reduced gut microbiota richness after surgery compared to control group (46). HD may require different lengths of surgical therapy depending on the degree of aganglionic involvement, which in turn may affect gut microbiota homeostasis (47). However, studies on the mechanism of GM underlying the development of HD were limited. Through our MR analyses, we found lines linking GM and HD by the mediation of metabolites.

The genus *Roseburia* belongs to the phylum *Firmicutes*, class *Clostridia*, order *Clostridiales*, and family *Lachnospiraceae (*48). There are five well-characterized *Roseburia* species: *Roseburia intestinalis*, *Roseburia hominis*, *Roseburia inulinivorans*, *Roseburia faecis*, and *Roseburia cecicola* (49). *Roseburia intestinalis* is known to protect against several inflammatory diseases by minimizing intestinal inflammatory reaction (48). The expression of colonic mucosal melatonin is positively related to *Roseburia hominis* (50). *Roseburia inulinivorans*, a newly discovered motile member of the *Firmicutes*, helps form butyrate from different dietary polysaccharide substrates present in the human large intestine (51). *Roseburia faecis,* a Gram-positive anaerobic bacterium that produces butyrate, has been tested for its usefulness in treating irritable bowel syndrome induced by repeated water avoidance stress in rat models (52). To date, no research has been carried out to explore the presence of the genus *Roseburia* in individuals with HD. Our MR analysis found that *Roseburia* may be involved in HD as a risk factor due to the mediation effect of serum X-21733. However, the information regarding serum X-21733 is unknown, and further molecular experiments are required to elucidate this mystery.

There has been debate regarding the precise role of *Peptococcus* in the human gut. Bourgault et al. conducted a study revealing *Peptococcus* as the most frequently occurring species of anaerobic Gram-positive cocci in significant infections (53). Moreover, it appears to be particularly pathogenic in infections of bones and joints or when present with foreign bodies (53). However, Gu et al. conducted research that demonstrated the protective effect of *Peptococcus* on inflammatory disorders of the breast (54). According to their study, there is a strong positive correlation between *Peptococcus* and the presence of valeric acid and butyrate, which regulate the inflammatory response and participate in the energy supply of tight junction proteins (54). Our MR analyses supported the protective effect of *Peptococcus* on HD, which may be mediated by the levels of stearoyl sphingomyelin (d18:1/18:0) and lysine. In the future, success may come from therapeutics targeting *Roseburia* or *Peptococcus*, including probiotics, fecal microbiota transplantation, prebiotics, and synbiotics.

Other bacteria were not associated with the development of HD. *Ruminococcus gnavus* is a Gram-positive strict anaerobe bacterium that forms chains. The bacteremia has been associated with an acute flare of ulcerative colitis (55). *Erysipelotrichaceae* has effect on the metabolism of lipid, warranting additional investigation into the metabolic profiles of these organisms. *Erysipelotrichaceae* may be correlated to inflammation and auto-immunogenic procedure (56).

Metabolomics is an advanced technology with great promise for uncovering the underlying mechanisms linked to various disease processes (57). Yang et al. identified 21 HD biomarkers in serum samples using metabolomic analysis (58). Sphingomyelin (d18:1/18:0) is a sphingolipid commonly found in cell membranes, particularly in the membranous myelin sheath surrounding nerve cell axons (59). Plekhova et al. discovered that lysine catabolism was increased in HD patients. This has been associated with inadequate bacterial butyrate production in the large bowel (60). Our study has found that serum levels of sphingomyelin (d18:1/18:0), lysine, and X-21733 were involved in mediating the GM-HD effect. However, the structure and function of X-21733 is still unknown. Our MR analyses have confirmed that metabolites are involved in the mechanism of HD development. To fully understand the underlying pathologies, further experiments are necessary.

This study has several advantages. Firstly, we used summary data from the GWAS consortium in our MR analyses, which increases statistical power since genetic variation is unaffected by confounding factors. Secondly, we combined bidirectional and two-step MR analyses to explore the potential mediation effects of metabolites, producing more precise and reliable results. This innovative approach provides a comprehensive understanding of the causal relationship between GM and HD.

There are a few limitations to consider in this study. Firstly, even if all three assumptions are fulfilled, it is impossible to avoid weak instrumental bias. However, GWAS data with larger sample sizes can help reduce this bias. Secondly, since the GWAS participants were only of European descent, it may not be appropriate to generalize the findings. Therefore, similar studies need to be conducted across multi-ethnic groups. Thirdly, MR analysis typically reveals exposure over a lifetime, and the presence of canalization may lead to an overestimation of effect size. Therefore, it is recommended that further randomized controlled trials be conducted to examine the effect.

## 5 Conclusions

In conclusion, our study indicated a causal relationship between *Peptococcus* and the incidence of HD that is partially mediated by serum levels of stearoyl sphingomyelin (d18:1/18:0) and lysine. Levels of *Peptococcus* in feces and stearoyl sphingomyelin (d18:1/18:0) and lysine levels in serum could serve as novel biomarkers and therapeutic targets for HD.

## Data Availability

The datasets presented in this study can be found in online repositories. The names of the repository/repositories and accession number(s) can be found in the article/Supplementary Material.
