# Supplementary material for: The mediating role of metabolites between gut microbiome and Hirschsprung disease: a bidirectional two-step Mendelian randomization study
Source: Front Pediatr. 2024 Aug 27;12:1371933. doi: 10.3389/fped.2024.1371933 (PMC11384983; doi:10.3389/fped.2024.1371933)
Supplement: Supplementary file 1 [file Datasheet1.pdf]

Supplementary Figures

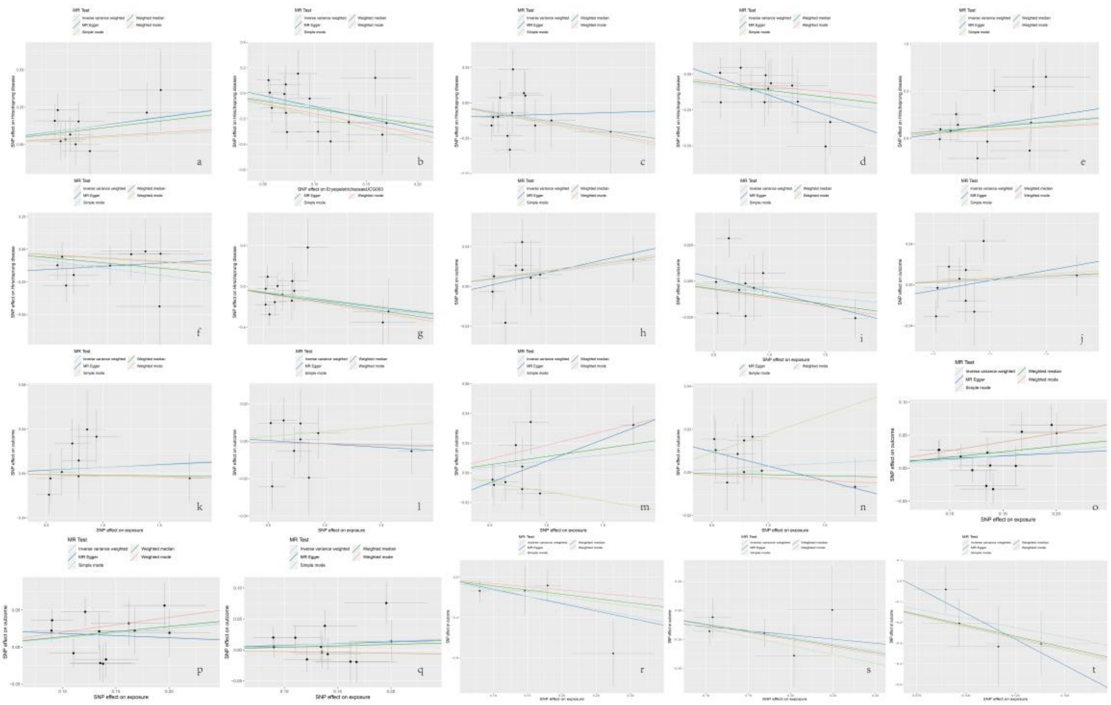

Figure S1. Scatter plots of our bidirectional, two-step MR analysis. (a) Effect of *Eggerthella* on HD; (b) Effect of *ErysipelotrichaceaeUCG003* on HD; (c) Effect of *Paraprevotella* on HD; (d) Effect of *Peptococcus* on HD; (e) Effect of *Roseburia* on HD; (f) Effect of *RuminococcaceaeNK4A214group* on HD; (g) Effect of *Ruminococcus2* on HD; (h) Effect of HD on *Eggerthell*; (i) Effect of HD on *ErysipelotrichaceaeUCG003*; (j) Effect of HD on *Paraprevotella*; (k) Effect of HD on *Peptococcus*; (l) Effect of HD on *Roseburia*; (m) Effect of HD on *RuminococcaceaeNK4A214group*; (n) Effect of HD on *Ruminococcus2*; (o) Effect of *Peptococcus* on Stearoyl sphingomyelin (d18:1/18:0) levels; (p) Effect of *Peptococcus* on Lysine levels; （q） Effect of *Roseburia* on X-21733 levels*;* (r) Effect of Stearoyl sphingomyelin (d18:1/18:0) levels on HD; (s) Effect of Lysine levels on HD; (t) Effect of X-21733 levels on HD.

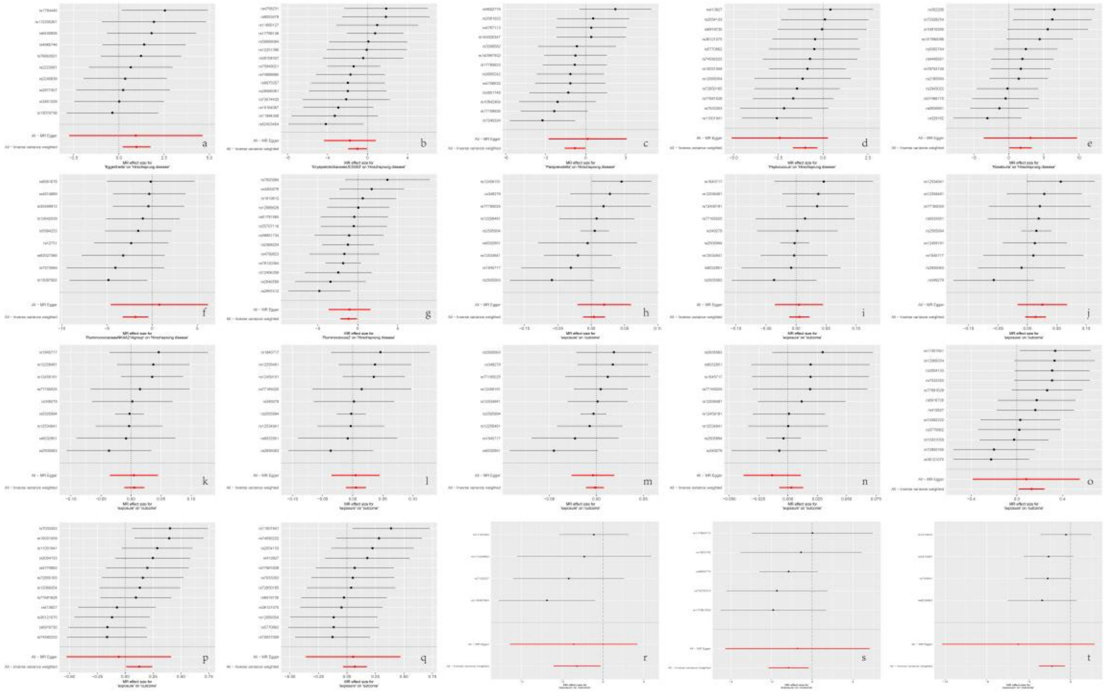

Figure S2. Forest plots of our bidirectional, two-step MR analysis. (a) Effect of *Eggerthella* on HD; (b) Effect of *ErysipelotrichaceaeUCG003* on HD; (c) Effect of *Paraprevotella* on HD; (d) Effect of *Peptococcus* on HD; (e) Effect of *Roseburia* on HD; (f) Effect of *RuminococcaceaeNK4A214group* on HD; (g) Effect of *Ruminococcus2* on HD; (h) Effect of HD on *Eggerthell*; (i) Effect of HD on *ErysipelotrichaceaeUCG003*; (j) Effect of HD on *Paraprevotella*; (k) Effect of HD on *Peptococcus*; (l) Effect of HD on *Roseburia*; (m) Effect of HD on *RuminococcaceaeNK4A214group*; (n) Effect of HD on *Ruminococcus2*; (o) Effect of *Peptococcus* on Stearoyl sphingomyelin (d18:1/18:0) levels; (p) Effect of *Peptococcus* on Lysine levels; （q） Effect of *Roseburia* on X-21733 levels*;* (r) Effect of Stearoyl sphingomyelin (d18:1/18:0) levels on HD; (s) Effect of Lysine levels on HD; (t) Effect of X-21733 levels on HD.

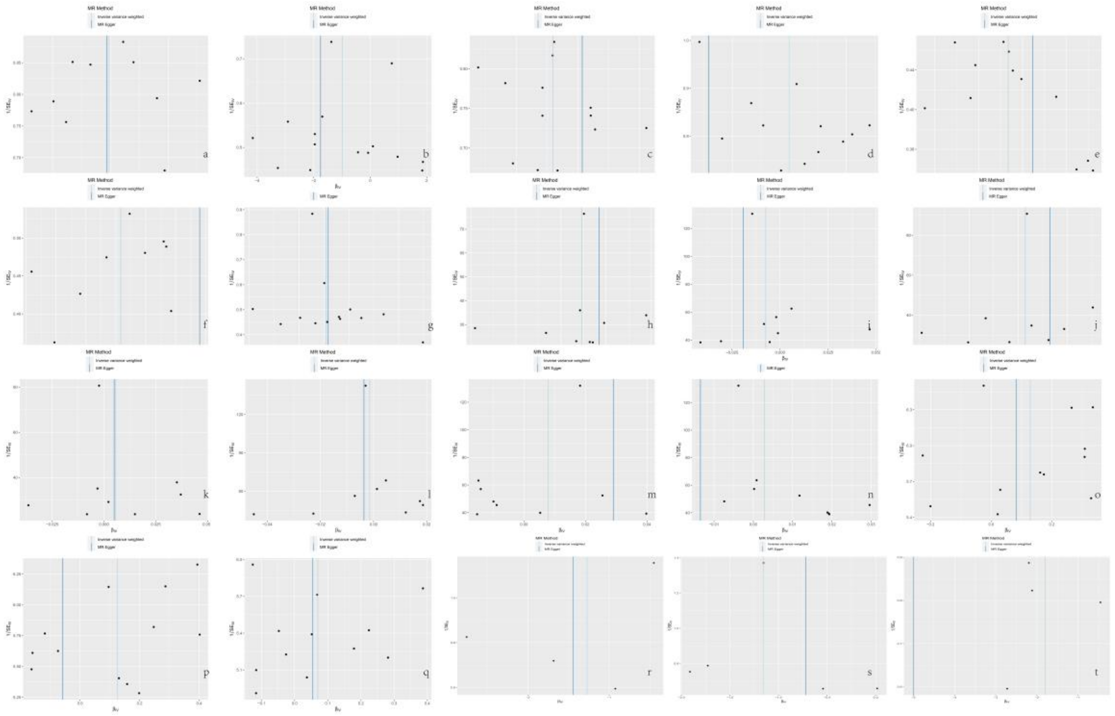

Figure S3. Funnel plots of our bidirectional, two-step MR analysis. (a) Effect of *Eggerthella* on HD; (b) Effect of *ErysipelotrichaceaeUCG003* on HD; (c) Effect of *Paraprevotella* on HD; (d) Effect of *Peptococcus* on HD; (e) Effect of *Roseburia* on HD; (f) Effect of *RuminococcaceaeNK4A214group* on HD; (g) Effect of *Ruminococcus2* on HD; (h) Effect of HD on *Eggerthell*; (i) Effect of HD on *ErysipelotrichaceaeUCG003*; (j) Effect of HD on *Paraprevotella*; (k) Effect of HD on *Peptococcus*; (l) Effect of HD on *Roseburia*; (m) Effect of HD on *RuminococcaceaeNK4A214group*; (n) Effect of HD on *Ruminococcus2*; (o) Effect of *Peptococcus* on Stearoyl sphingomyelin (d18:1/18:0) levels; (p) Effect of *Peptococcus* on Lysine levels; （q） Effect of *Roseburia* on X-21733 levels; (r) Effect of Stearoyl sphingomyelin (d18:1/18:0) levels on HD; (s) Effect of Lysine levels on HD; (t) Effect of X-21733 levels on HD.

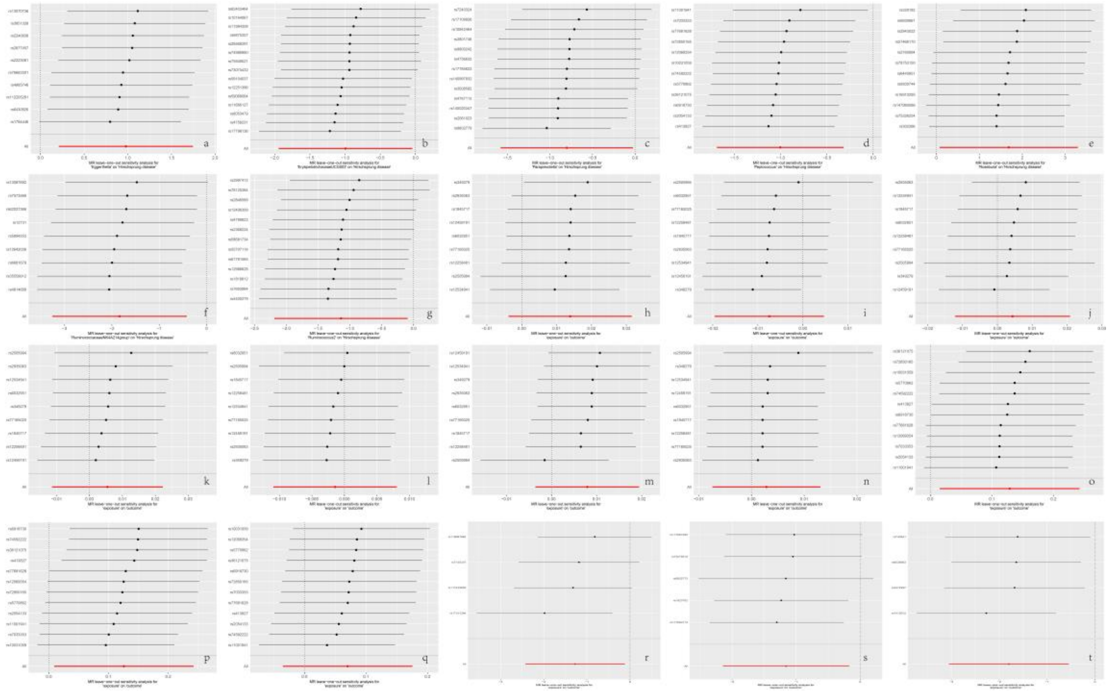

Figure S4. Leave-one-out plots of our bidirectional, two-step MR analysis. (a) Effect of *Eggerthella* on HD; (b) Effect of *ErysipelotrichaceaeUCG003* on HD; (c) Effect of *Paraprevotella* on HD; (d) Effect of *Peptococcus* on HD; (e) Effect of *Roseburia* on HD; (f) Effect of *RuminococcaceaeNK4A214group* on HD; (g) Effect of *Ruminococcus2* on HD; (h) Effect of HD on *Eggerthell*; (i) Effect of HD on *ErysipelotrichaceaeUCG003*; (j) Effect of HD on *Paraprevotella*; (k) Effect of HD on *Peptococcus*; (l) Effect of HD on *Roseburia*; (m) Effect of HD on *RuminococcaceaeNK4A214group*; (n) Effect of HD on *Ruminococcus2*; (o) Effect of *Peptococcus* on Stearoyl sphingomyelin (d18:1/18:0) levels; (p) Effect of *Peptococcus* on Lysine levels; （q） Effect of *Roseburia* on X-21733 levels; (r) Effect of Stearoyl sphingomyelin (d18:1/18:0) levels on HD; (s) Effect of Lysine levels on HD; (t) Effect of X-21733 levels on HD.
