# Supplementary material for: The mediating role of metabolites between gut microbiome and Hirschsprung disease: a bidirectional two-step Mendelian randomization study
Source: Front Pediatr. 2024 Aug 27;12:1371933. doi: 10.3389/fped.2024.1371933 (PMC11384983; doi:10.3389/fped.2024.1371933)
Supplement: Supplementary file 3 [file Table1.pdf]

Table S1. Forward MR analysis of GM on HD.

Outcome	Exposure	Method	nSNP	beta	se	pval	lo_ci	up_ci	or	or_lci95	or_uci95	Egger-intercept	Egger-pval	Q	Q_pval
HD	Peptococcus	MR Egger	12	-2.421	1.374	0.109	-5.113	0.272	0.089	0.006	1.312	0.189	0.311	6.94	0.804
		Weighted median	12	-0.816	0.473	0.085	-1.743	0.112	0.442	0.175	1.118				
		Inverse variance weighted	12	-1.004	0.35	0.004	-1.69	-0.318	0.366	0.185	0.727				
		Simple mode	12	-0.622	0.856	0.483	-2.299	1.056	0.537	0.1	2.874				
		Weighted mode	12	-0.622	0.797	0.452	-2.184	0.94	0.537	0.113	2.56				
HD	Ruminococcaceae NK4A214group	MR Egger	9	0.806	2.749	0.778	-4.583	6.194	2.238	0.01	489.924	-0.192	0.352	4.589	0.8
		Weighted median	9	-1.327	0.97	0.171	-3.228	0.574	0.265	0.04	1.775				
		Inverse variance weighted	9	-1.838	0.724	0.011	-3.258	-0.419	0.159	0.038	0.658				
		Simple mode	9	-0.827	1.398	0.57	-3.567	1.912	0.437	0.028	6.768				
		Weighted mode	9	-0.89	1.361	0.532	-3.558	1.778	0.411	0.028	5.917				
HD	Eggerthella	MR Egger	10	0.936	1.882	0.632	-2.753	4.626	2.55	0.064	102.069	0.005	0.982	5	0.834
		Weighted median	10	0.837	0.537	0.119	-0.216	1.89	2.309	0.805	6.619				
		Inverse variance weighted	10	0.978	0.392	0.013	0.21	1.747	2.66	1.234	5.737				
		Simple mode	10	0.365	0.844	0.675	-1.289	2.02	1.441	0.275	7.537				
		Weighted mode	10	0.425	0.788	0.602	-1.119	1.969	1.53	0.327	7.164				
HD	Ruminococcus2	MR Egger	13	-1.028	1.34	0.459	-3.653	1.598	0.358	0.026	4.943	-0.01	0.928	11.61	0.477
		Weighted median	13	-1.248	0.724	0.085	-2.667	0.172	0.287	0.069	1.187				
		Inverse variance weighted	13	-1.141	0.535	0.033	-2.19	-0.092	0.32	0.112	0.912				
		Simple mode	13	-1.029	1.165	0.395	-3.313	1.256	0.358	0.036	3.51				
		Weighted mode	13	-1.358	1.012	0.204	-3.341	0.625	0.257	0.035	1.868				

HD	Erysipelotrichaceae UCG003	MR Egger	15	-1.756	1.321	0.207	-4.345	0.833	0.173	0.013	2.3	0.07	0.546	12.26	0.585
		Weighted median	15	-1.219	0.68	0.073	-2.551	0.113	0.295	0.078	1.119				
		Inverse variance weighted	15	-0.995	0.485	0.04	-1.946	-0.044	0.37	0.143	0.957				
		Simple mode	15	-1.807	1.16	0.142	-4.081	0.467	0.164	0.017	1.595				
		Weighted mode	15	-1.598	1.145	0.185	-3.844	0.647	0.202	0.021	1.909				
HD	Roseburia	MR Egger	12	3.089	3.408	0.386	-3.592	9.769	21.944	0.028	17476.21	-0.095	0.679	15.42	0.164
		Weighted median	12	1.612	1.001	0.108	-0.351	3.575	5.012	0.704	35.686				
		Inverse variance weighted	12	1.684	0.822	0.04	0.074	3.294	5.387	1.076	26.96				
		Simple mode	12	1.244	1.705	0.481	-2.098	4.585	3.469	0.123	98.035				
		Weighted mode	12	1.128	1.485	0.463	-1.783	4.039	3.089	0.168	56.752				
HD	Paraprevotella	MR Egger	13	0.145	1.5	0.925	-2.796	3.086	1.156	0.061	21.884	-0.104	0.525	13.79	0.314
		Weighted median	13	-0.808	0.519	0.12	-1.826	0.21	0.446	0.161	1.234				
		Inverse variance weighted	13	-0.802	0.397	0.043	-1.58	-0.024	0.449	0.206	0.977				
		Simple mode	13	-0.949	0.928	0.327	-2.767	0.87	0.387	0.063	2.386				
		Weighted mode	13	-0.889	0.874	0.329	-2.602	0.823	0.411	0.074	2.277				

HD: Hirschsprung disease; IVW: inverse-variance weighted; nSNP: number of single nucleotide polymorphisms; SE: standard error; or: odds ratio.

Table S2. Reverse MR analysis of HD on GM.

Exposure	Outcome	Method	nSNP	beta	SE	pval	or	or_lci95	or_uci95	Egger-intercept	Egger-pval	Q	Q_pval
HD	*Ruminococcus2*	IVW	9	0.003	0.005	0.586	1.003	0.993	1.013	0.016	0.193	3.996	0.857
HD	*ErysipelotrichaceaeUCG003*	IVW	9	-0.007	0.006	0.229	0.993	0.981	1.005	0.012	0.419	10.88	0.208
HD	*Roseburia*	IVW	9	-0.001	0.005	0.766	0.999	0.989	1.008	0.002	0.842	6.803	0.558
HD	*Paraprevotella*	IVW	9	0.004	0.008	0.606	1.004	0.988	1.021	-0.015	0.442	9.688	0.288
HD	*Peptococcus*	IVW	9	0.005	0.009	0.525	1.005	0.989	1.023	0.0006	0.976	5.283	0.727
HD	*RuminococcaceaeNK4A214group*	IVW	9	0.008	0.006	0.177	1.008	0.996	1.02	-0.021	0.104	10.05	0.262
HD	*Eggerthella*	IVW	9	0.014	0.009	0.124	1.014	0.996	1.032	-0.012	0.574	6.882	0.549

HD: Hirschsprung disease; IVW: inverse-variance weighted; nSNP: number of single nucleotide polymorphisms; SE: standard error; or: odds ratio.

Table S3. MR analysis of significant GM on metabolites.

Exposure	Outcome	nSNP	beta	SE	pval	or	or_lci95	or_uci95	Egger-intercept	Egger-pval	Q	Q_pval
Roseburia	X-21733 levels	13	-0.22	0.089	0.013	0.803	0.675	0.955	0.002	0.94	9.371	0.587
Peptococcus	Stearoyl sphingomyelin (d18:1/18:0) levels	12	0.129	0.058	0.026	1.137	1.015	1.274	0.006	0.843	15.38	0.166
Peptococcus	Lysine levels	12	0.126	0.06	0.035	1.134	1.009	1.275	0.024	0.445	15.43	0.163

nSNP: number of single nucleotide polymorphisms; SE: standard error; or: odds ratio.

Table S4. MR analysis of significant metabolites on HD.

Exposure	Outcome	pvalue	nSNP	beta	SE	pval	or	or_lci95	or_uci95	Egger-intercept	Egger-pval	Q	Q_pval
3-hydroxybutyroylglycine levels	HD	0.008	6	-1.57	0.589	0.008	0.208	0.066	0.659	0.079	0.729	1.536	0.909
Glutamine conjugate of C6H10O2 (1) levels	HD	0.004	3	-2.356	0.828	0.004	0.095	0.019	0.48	0.007	0.987	0.176	0.916
Stearoyl sphingomyelin (d18:1/18:0) levels	HD	0.03	4	-1.273	0.588	0.03	0.28	0.088	0.887	0.026	0.918	2.522	0.471
Lysine levels	HD	0.022	5	-1.158	0.504	0.022	0.314	0.117	0.844	-0.067	0.817	1.285	0.864
Glucuronide of piperine metabolite C17H21NO3 (3) levels	HD	0.024	3	2.057	0.908	0.024	7.822	1.319	46.396	-0.12	0.702	0.611	0.737
Mannose levels	HD	0.018	5	-1.567	0.66	0.018	0.209	0.057	0.761	0.142	0.918	0.605	0.963
16a-hydroxy DHEA 3-sulfate levels	HD	0.019	4	-1.654	0.705	0.019	0.191	0.048	0.761	-0.092	0.763	1.768	0.622
X-25371 levels	HD	0.001	3	1.592	0.484	0.001	4.912	1.901	12.691	-0.113	0.679	0.36	0.835
Arachidonate (20:4n6) to caffeine ratio	HD	0.029	8	1.108	0.508	0.029	3.027	1.119	8.192	0.384	0.123	6.631	0.468
3-methylxanthine levels	HD	0.035	5	-1.35	0.639	0.035	0.259	0.074	0.908	-0.075	0.795	2.706	0.608
Theophylline levels	HD	0.031	7	-1.184	0.548	0.031	0.306	0.105	0.896	-0.148	0.346	3.927	0.687
2,3-dihydroxypyridine levels	HD	0.031	3	1.875	0.867	0.031	6.52	1.192	35.673	0.071	0.881	1.604	0.448
EDTA levels	HD	0.05	5	1.31	0.669	0.05	3.708	1	13.748	-0.173	0.617	2.074	0.722
Caproate (6:0) levels	HD	0.035	5	1.385	0.656	0.035	3.996	1.105	14.446	0.613	0.212	3.084	0.544
X-21733 levels	HD	0.005	4	-1.8	0.639	0.005	0.165	0.047	0.578	0.344	0.475	1.638	0.651
Glycine levels	HD	0.015	4	-1.838	0.753	0.015	0.159	0.036	0.696	0.228	0.518	2.919	0.404

X-19141 levels	HD	0.049	3	1.812	0.919	0.049	6.123	1.012	37.059	0.342	0.753	0.224	0.894
Phosphate to 2'-deoxyuridine ratio	HD	0.04	7	1.104	0.538	0.04	3.016	1.052	8.649	0.021	0.907	3.393	0.758
2-palmitoleoyl-GPC (16:1) levels	HD	0.038	4	1.345	0.649	0.038	3.837	1.075	13.699	0.486	0.231	3.185	0.364
Isobutyrylglycine levels	HD	0.013	8	-1.235	0.495	0.013	0.291	0.11	0.767	0.211	0.155	7.323	0.396
X-12849 levels	HD	0.048	3	-1.869	0.945	0.048	0.154	0.024	0.983	0.123	0.778	0.377	0.828
X-11880 levels	HD	0.04	4	1.29	0.628	0.04	3.633	1.06	12.445	-0.125	0.615	2.281	0.516
X-12407 levels	HD	0.003	3	2.327	0.772	0.003	10.245	2.255	46.546	0.052	0.918	0.312	0.856
Inosine to theophylline ratio	HD	0.016	5	1.331	0.552	0.016	3.785	1.284	11.158	0.286	0.175	4.65	0.325
Mannose to fructose ratio	HD	0.04	4	-1.51	0.735	0.04	0.221	0.052	0.933	0.086	0.746	0.689	0.876
Cortisone to 4-cholesten-3-one ratio	HD	0.024	5	1.444	0.637	0.024	4.237	1.215	14.782	0.195	0.271	3.25	0.517
Citrate to 4-hydroxyphenylpyruvate ratio	HD	0.02	3	-1.696	0.728	0.02	0.183	0.044	0.764	0.176	0.61	0.519	0.771

HD: Hirschsprung disease; nSNP: number of single nucleotide polymorphisms; SE: standard error; or: odds ratio.
